# Diagnostic Value of Dual Energy Technology of Dual Source CT in Differentiation Grade of Colorectal Cancer

**DOI:** 10.2174/0115734056360004250828115402

**Published:** 2025-09-09

**Authors:** Sudhir K. Yadav, Nan Deng, Jikong Ma, Yixin Liu, Chunmei Zhang, Ling Liu

**Affiliations:** 1 Department of Radiology, The First Affiliated Hospital of Dali University, Dali 671000, China; 2 Department of Ultrasound Medicine, The Second Affiliated Hospital of Chengdu Medical College, 416 Hospital of Nuclear Industry, Sichuan, Chengdu, China; 3 Department of Nephrology, Dali Bai Autonomous Prefecture People’s Hospital, Dali 671000, China; 4 Department of Pathology, The First Affiliated Hospital of Dali University, Dali 671000, China

**Keywords:** Dual-energy CT, Colorectal cancer, Normalized iodine concentration, Differentiation grade, Dual-energy index, CRC

## Abstract

**Introduction:**

Colorectal cancer (CRC) is a leading cause of cancer-related morbidity and mortality. Accurate differentiation of tumor grade is crucial for prognosis and treatment planning. This study aimed to evaluate the diagnostic value of dual-source CT dual-energy technology parameters in distinguishing CRC differentiation grades.

**Methods:**

A retrospective analysis was conducted on 87 surgically and pathologically confirmed CRC patients (64 with medium-high differentiation and 23 with low differentiation) who underwent dual-source CT dual-energy enhancement scanning. Normalized iodine concentration (NIC), spectral curve slope (K), and dual-energy index (DEI) of the tumor center were measured in arterial and venous phases. Differences in these parameters between differentiation groups were compared, and ROC curve analysis was performed to assess diagnostic efficacy.

**Results:**

The low-differentiation group exhibited significantly higher NIC, K, and DEI values in both arterial and venous phases compared to the medium-high differentiation group (P < 0.01). In the arterial phase, NIC, K, and DEI yielded AUC values of 0.920, 0.770, and 0.903, respectively, with sensitivities of 95.7%, 65.2%, and 91.3%, and specificities of 82.8%, 75.0%, and 75.0%, respectively. In the venous phase, AUC values were 0.874, 0.837, and 0.886, with sensitivities of 91.3%, 82.6%, and 91.3%, and specificities of 68.75%, 75.0%, and 73.4%. NIC in the arterial phase showed statistically superior diagnostic performance compared to K values (P < 0.05).

**Discussion:**

Dual-energy CT parameters, particularly NIC in the arterial phase, demonstrate high diagnostic accuracy in differentiating CRC grades. These findings suggest that quantitative dual-energy CT metrics can serve as valuable non-invasive tools for tumor characterization, aiding in clinical decision-making. Study limitations include its retrospective design and relatively small sample size.

**Conclusion:**

NIC, K, and DEI values in dual-energy CT scans are highly effective in distinguishing CRC differentiation grades, with arterial-phase NIC showing the highest diagnostic performance. These parameters may enhance preoperative assessment and personalized treatment strategies for CRC patients.

## 1 INTRODUCTION

Colorectal cancer (CRC) is the most common malignant tumor globally, with significant regional disparities in incidence and mortality. According to GLOBOCAN 2023, CRC accounts for approximately 2.1 million new cases (10.2% of all cancers) and 1.1 million deaths annually, reflecting a rising burden compared to 2020 estimates. In China, CRC ranks second in incidence (13.4%) and third in mortality (9.7%), underscoring its public health urgency [1, 2]. Histological grading, endorsed by both the World Health Organization (WHO) and the American Joint Committee on Cancer (AJCC), remains a critical prognostic factor and therapeutic determinant for CRC patients [1].

Currently, Multi-Detector Computed Tomography (MDCT) is the cornerstone of preoperative CRC evaluation, offering widespread utility in staging, treatment monitoring, and follow-up [3, 4]. However, MDCT is limited by its reliance on morphological criteria (e.g., tumor size, wall thickness, and lymph node enlargement), which lack sensitivity in assessing tumor differentiation or molecular heterogeneity [5]. Recent studies have highlighted additional shortcomings, including beam-hardening artifacts, limited soft-tissue contrast, and inadequate spectral resolution for quantitative analysis [6, 7].

In contrast, Dual-Source CT (DSCT) dual-energy imaging overcomes these limitations by enabling material decomposition and multiparametric quantification. Graser *et al*. (2023) demonstrated that DSCT’s dual-energy mode improves lesion characterization through iodine mapping (reflecting perfusion), spectral curve slope (K, indicating tissue attenuation dynamics), and Dual-Energy Index (DEI, a marker of material-specific attenuation) [8]. These parameters correlate with tumor angiogenesis, cellular density, and glycolytic activity—features closely linked to differentiation grade [9, 10]. Notably, DSCT reduces metal artifacts and enhances contrast resolution, facilitating earlier detection of subtle CRC heterogeneity [11].

This study is the first to combine three DSCT-derived parameters—Normalized Iodine Concentration (NIC), K, and DEI—for noninvasive CRC differentiation grading. By integrating functional (NIC), dynamic (K), and compositional (DEI) data, the aim of this study is to establish a comprehensive imaging biomarker system that surpasses conventional MDCT’s anatomical constraints. Our approach aligns with the growing demand for precision oncology tools to guide personalized CRC management.

## 2 MATERIALS AND METHODS

### 2.1 General Information

A retrospective selection of 87 CRC patients, confirmed by surgical pathology and who underwent DSCT dual-energy enhanced scans preoperatively between December 2018 and December 2021, was made. Of these, there were 38 female and 49 male patients, aged between 36 and 87 years. There were 68 cases of rectal cancer and 19 cases of colon cancer. The main clinical symptoms included lower abdominal discomfort, abdominal pain, bloody stools, changes in stool characteristics, and loss of appetite.

### 2.2 Ethical Approval

This study was approved by the First Affiliated Hospital of Dali Universit*y,* Institutional Review Ethics Committee, cited as Approval No. DFY20250526001. Patient consent was waived due to the retrospective nature of the study.

### 2.3 Equipment and Methods

All studies were performed using a Siemens Somatom Force third-generation dual-source CT scanner, with full-abdominal plain and dual-energy enhanced scans. Patients were instructed to fast for 6–8 hours before the examination and to drink 500–1000 ml of water. The routine scanning position was supine, and the scanning range extended from the top of the diaphragm to the level of the symphysis pubis. The scanning parameters were as follows: collimator specifications of 192×0.6mm, tube rotation time of 0.5s, pitch of 0.6, slice thickness of 5mm, tube voltage of 90kVp, and tube current of 220mAs. A MedradStellant dual-head injector was used to inject iopamidol (300mgI/ml, Yangtze River Pharmaceutical Group Co., Ltd.) into the patient's elbow vein at a flow rate of 3ml/s and a dose of 1.5ml/kg. Arterial phase scanning was initiated 7 seconds after the abdominal aorta reached a threshold of 100HU, and venous phase scanning was performed 25 seconds later. The A tube had a voltage of 100kV and a current limit of 180As, while the B tube had a voltage of 150kV and a current limit of 90As, with CARE Dose 4D activated. Images obtained from the scans were transmitted to the Picture Archiving and Communication System (PACS) and Siemens post-processing software for further analysis.

### 2.4 Image Post-Processing

Using the Siemens syngo MMWP VE36A workstation, dual-energy arterial and venous phase 1mm thin-slice images were loaded. Two experienced radiologists measured the Region of Interest (ROI) to obtain DEI values. The “Liver VNC” mode in the post-processing software was used to acquire iodine maps, from which the iodine values in the ROI were measured. The “Mono Energetic” mode was used for spectral analysis, and CT value variations at 40keV to 190keV in the ROI were measured to obtain the spectral curve. The ROI was selected at the most enhanced part of the primary colorectal lesion, with a size of approximately 0.3–0.6cm^2^, avoiding blood vessels and necrotic areas. Measurements were taken three times on the same layer, with the average taken. NIC was calculated as the lesion iodine value divided by the iodine value in the blood vessel on the same layer. The slope of the spectral curve (K) was calculated as K=(Hu40keV-Hu100keV)/60, and the NIC, K, and DEI values for both arterial and venous phase ROIs were obtained.

### 2.5 Statistical Analysis

Statistical analysis was performed using SPSS 25.0 software. The data in this study were normally distributed. Quantitative data were described as mean ± standard deviation (x̄±s), and independent sample t-tests were used to compare NIC, K, and DEI between the two groups. A P-value < 0.05 was considered statistically significant. Receiver Operating Characteristic (ROC) curves were drawn to calculate the sensitivity, specificity, optimal diagnostic threshold, and Area Under the Curve (AUC) for NIC, K, and DEI in both arterial and venous phases for diagnosing different degrees of CRC differentiation. The diagnostic efficacy of each index was analyzed, and MedCalc 19.1 software was used to compare AUC values between parameters.

## 3 RESULTS

Pathological results indicated that all 87 cases of CRC were adenocarcinomas. The cases were divided into two groups based on the degree of differentiation: Group 1 (moderately to well-differentiated, 64 cases, including 56 moderately differentiated and 8 well-differentiated cases) and Group 2 (poorly differentiated, 23 cases) (Table **1**).

### 3.1 Comparison of Dual-Energy Parameters in Different CRC Differentiation Levels During Arterial and Venous Phases

**Fig. (1):**
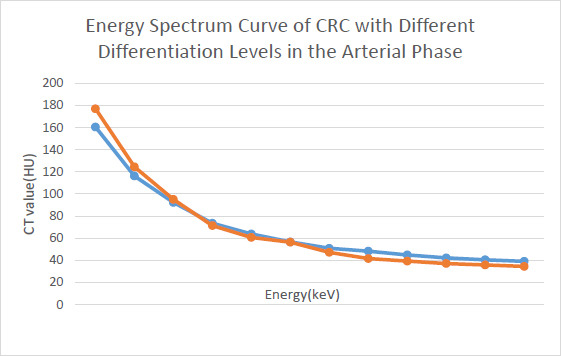
Comparison of Energy Spectrum Curves of Moderately/Well-Differentiated CRC and Poorly Differentiated CRC during the Arterial Phase. **Legends:** *Red*: Poorly differentiated (steeper slope, K=2.31). *Blue*: Moderately/well-differentiated.

**Fig. (2):**
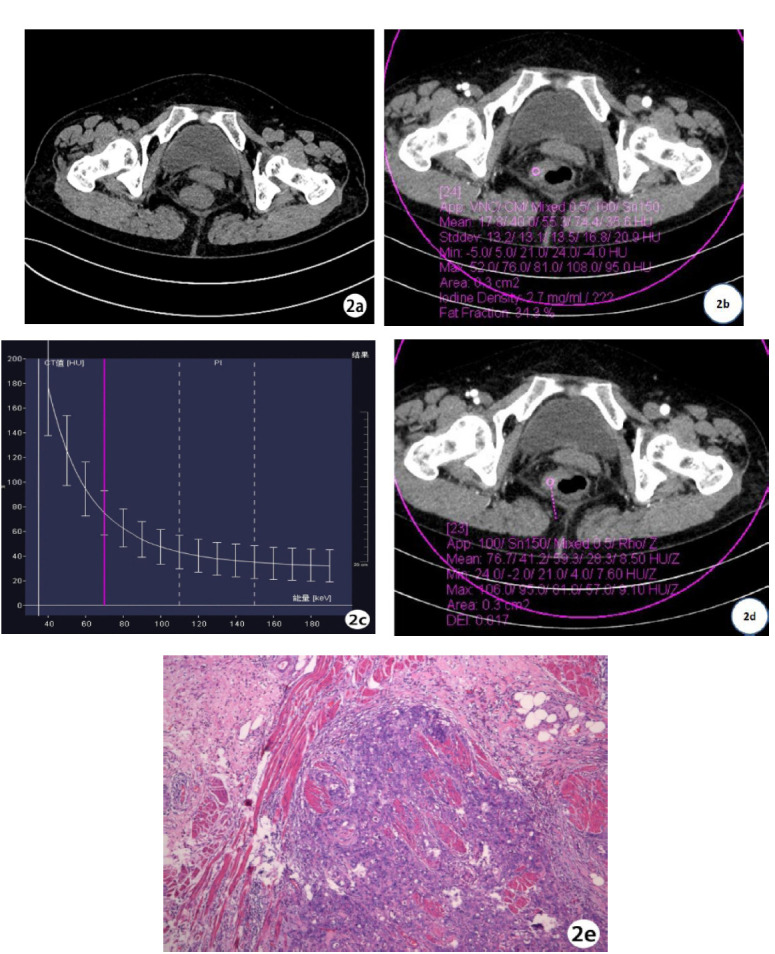
Female, 59 years old, with poorly differentiated rectal cancer. (**a**) shows irregular thickening of the rectal wall in a plain scan image; (**b**) shows the iodine concentration of 2.7mg/ml in arterial phase lesions; The energy spectrum curve in (**c**) shows that the K value of the arterial phase lesion is 2.38; (**d**) dual energy image shows a DEI of 0.017 for arterial phase lesions; (**e**) shows a poorly differentiated adenocarcinoma (HE × 100) of the rectum, with almost no glandular ducts or acinar structures.

**Fig. (3):**
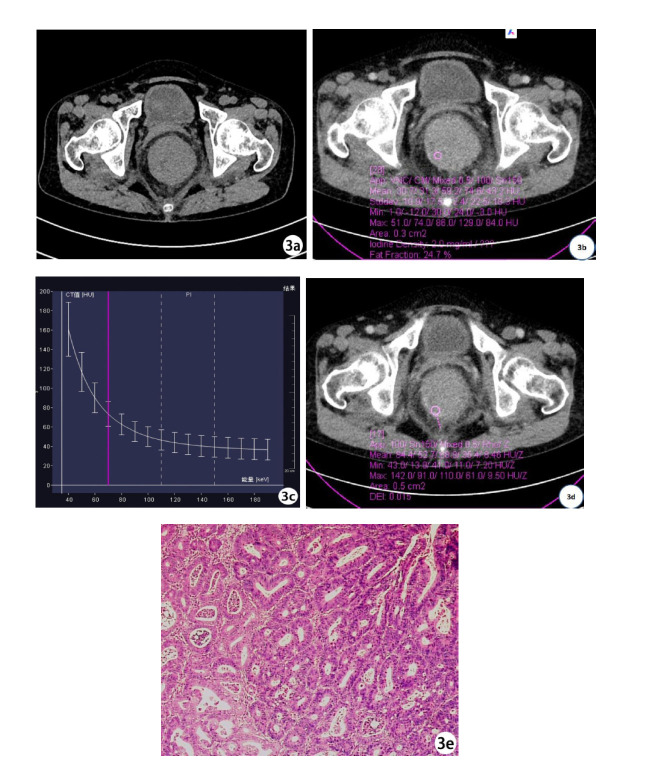
Female, 61 years old, with moderately differentiated rectal cancer. (**a**) shows a plain scan image with significant thickening of the rectal wall and narrowing of the lumen; (**b**) shows the iodine concentration of 2.0mg/ml in arterial phase lesions; The energy spectrum curve in (**c**) shows that the K value of the arterial phase lesion is 2.02 (**d**); The 3D dual energy image shows a DEI of 0.015 for the arterial phase lesion; (**e**) shows a moderately differentiated adenocarcinoma (HE × 100) in the rectum, with visible glandular ducts and acinar structures.

In the arterial phase, NIC, K, and DEI values were higher in the poorly differentiated CRC group than in the moderately to well-differentiated CRC group. Both groups showed a downward spectral curve; the lower the differentiation, the steeper the curve (Figs. **1**, **2a**-**e** and **3a**-**e**). Statistical data showed that NIC, K, and DEI differences between the two groups were statistically significant (P < 0.01). In the venous phase, NIC, K, and DEI values were also higher in the poorly differentiated CRC group, and both groups exhibited downward spectral curves, with the poorly differentiated group showing steeper curves. Statistical data again showed significant differences between the two groups (P < 0.01) (Table **2**).

### 3.2 Diagnostic Efficacy of Different Dual-Energy Parameters in Arterial and Venous Phases for Differentiation of CRC

**Fig. (4):**
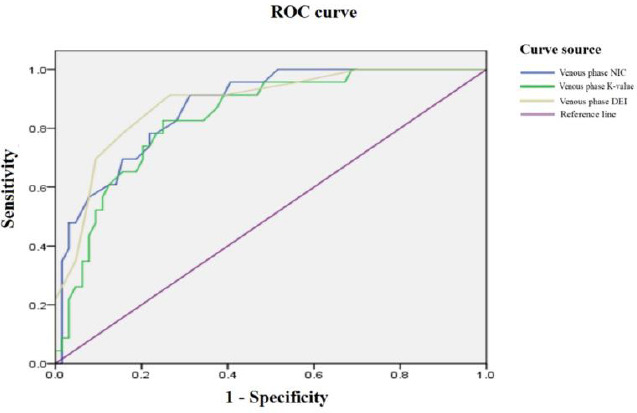
Arterial Phase ROC Curves of NIC, K Value, and DEI for diagnosing differentiation of CRC.

**Fig. (5):**
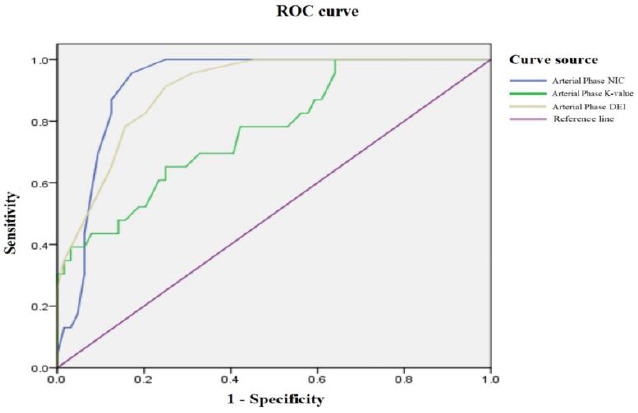
Venous phase ROC Curves of NIC, K Value, and DEI for diagnosing differentiation of CRC.

The ROC curves of different parameters show that the AUCs of NIC, K value, and DEI during the arterial phase were 0.920, 0.770, and 0.903, respectively (Fig. **4**). The sensitivities are 95.7%, 65.2%, and 91.3%, and the specificities are 82.8%, 75.0%, and 75.0%, with diagnostic thresholds of 0.255 mg/mL, 2.035, and 0.0205, respectively (Table **3**). During the venous phase, the AUCs of NIC, K value, and DEI were 0.874, 0.837, and 0.886, respectively (Fig. **5**), with sensitivities of 91.3%, 82.6%, and 91.3%, and specificities of 68.75%, 75.0%, and 73.4%. The diagnostic thresholds were 0.705 mg/mL, 2.17, and 0.0245, respectively (Table **4**). Among these, the AUC of NIC in the arterial phase was higher than other parameters. Comparing the AUC of NIC in the arterial phase with other parameters, the difference was statistically significant (Z=2.055, p=0.0399). There were no statistically significant differences between other parameters and NIC in the arterial phase (p>0.05).

## 4 DISCUSSION

Colorectal cancer (CRC) is the third most common malignancy worldwide and a major cause of cancer-related deaths [1]. Tumor differentiation plays a crucial role in patient prognosis, as poorly differentiated tumors tend to be more malignant, invasive, and associated with worse outcomes [2]. Accurate preoperative grading is vital for formulating appropriate treatment strategies and predicting disease progression [3].

Dual-Source Computed Tomography (DSCT) dual-energy imaging uses two X-ray energy levels to scan tissues, leveraging differences in X-ray attenuation to distinguish tissue components [4]. Iodine concentration (IC), derived from iodine maps, reflects lesion enhancement and minimizes variability caused by factors like tube voltage, contrast agent dose, and patient size [5, 6]. Our findings demonstrate that normalized iodine concentration (NIC) is significantly higher in poorly differentiated tumors compared to moderately to highly differentiated tumors during both arterial and venous phases (p<0.01) [7, 8]. This aligns with the understanding that poorly differentiated tumors exhibit more aggressive biological behavior, characterized by rapid angiogenesis and richer blood supply [9, 10]. The lower NIC in the arterial phase compared to the venous phase can be attributed to gradual contrast agent permeation into the extracellular matrix [11].

The spectral curve slope has also shown promise in diagnosing tumor differentiation levels. In this study, K values and Dual-Energy Index (DEI) were significantly different between poorly differentiated and moderately to highly differentiated groups (p<0.01) [12, 13]. Poorly differentiated tumors exhibited steeper spectral curves and higher K values, consistent with structural differences observed in prior research [14, 15]. The DEI, calculated as (HU80KeV - HU140KeV)/(HU80KeV + HU140KeV + 2000), was also higher in the poorly differentiated group [16]. These quantitative parameters offer a non-invasive method for preoperative CRC grading, with advantages including shorter scanning times and reduced radiation exposure compared to traditional CT [17].

Previous studies have established NIC thresholds for CRC differentiation. Gong *et al*. reported that poorly differentiated tumors had significantly higher arterial-phase NIC values (>0.25 mg/mL) compared to well-differentiated tumors (<0.18 mg/mL) [18]. While our findings corroborate this pattern, slightly lower thresholds (>0.22 mg/mL) were identified, possibly due to differences in: (1) contrast injection protocols, (2) arterial phase timing, and (3) ROI placement methodology [15]. Similarly, while Al-Najami *et al*. reported wider DEI variability in poorly differentiated CRC [17], our results show that DEI remained consistently higher in these tumors (p<0.01), with the spectral curve slope (K) demonstrating less dispersion than DEI [19].

Key contributions of this study include: (1) demonstration of arterial-phase NIC's superior diagnostic performance (AUC 0.920 vs venous-phase 0.870), likely due to better capture of early angiogenic dynamics; (2) validation of spectral curve slope as a robust complementary metric; and (3) potential for machine learning integration to enhance diagnostic accuracy through multi-parametric analysis [20].

Several limitations should be noted: (1) single-center design may limit generalizability; (2) manual ROI placement could introduce selection bias; (3) temporal factors in arterial-phase timing may affect measurements; and (4) histopathologic overlap may cause diagnostic ambiguity [21]. Future directions should include multi-center validation studies, AI-powered automated analysis, and exploration of molecular marker correlations to refine prognostic stratification [22], including potential synergies between imaging biomarkers and serum markers like butyrylcholinesterase for postoperative monitoring [23].

Recent advances in AI algorithms, particularly convolutional neural networks (CNNs), have shown promise in automating CRC grading from CT images [24]. The integration of DECT parameters with such AI approaches can significantly reduce inter-observer variability, though multicenter validation trials are needed to establish clinical utility [24]. Beyond imaging biomarkers, emerging serum markers like butyrylcholinesterase have demonstrated clinical value, with elevated postoperative levels correlating with anastomotic leaks [25], suggesting potential utility in combination with imaging biomarkers for comprehensive patient monitoring

## CONCLUSION

Dual-energy parameters derived from DSCT provide robust support for preoperative grading and valuable insights into the differentiation levels of CRC. The potential of DECT as a non-invasive diagnostic technique is highlighted by the notable variations in NIC, K values, and DEI between tumors that are poorly differentiated and those that are moderately to highly differentiated. In order to further improve diagnostic accuracy, future research should concentrate on confirming these results in larger cohorts and investigating the incorporation of AI. AI and DECT together have the potential to completely transform the preoperative evaluation of colorectal cancer and enhance patient outcomes in the long run.

## Figures and Tables

**Table 1:** Demographic profile of CRC patients.

**Characteristic**	**Moderately/Well-Differentiated (n=64)**	**Poorly Differentiated (n=23)**
Age (years)	58.2 ± 10.5	62.4 ± 9.8
Sex (Male/Female)	35/29	14/9
Tumor location (Colon/Rectum)	12/52	7/16

**Table 2:** Comparison of NIC, K value, and DEI in different differentiation levels of CRC during arterial and venous phases.

**Group**	**Differentiation Level**	**Number of Cases**	**NIC**		**K value**		**DEI**	
			**(Arterial Phase)**	**(Venous Phase)**	**(Arterial Phase)**	**(Venous Phase)**	**(Arterial Phase)**	**(Venous Phase)**
1	Moderately/well differentiated	64	0.23±0.04	0.66±0.12	1.82±0.26	2.03±0.24	0.018±0.003	0.023±0.003
2	Poorly differentiated	23	0.30±0.03	0.83±0.11	2.13±0.28	2.31±0.17	0.024±0.003	0.028±0.004
t-value	-	-	-7.374	-5.916	-4.688	-5.281	-7.456	-7.284
p-value	-	-	0.000	0.000	0.000	0.000	0.000	0.000

**Table 3:** ROC curve analysis of NIC, K-value, and DEI for diagnosing differentiation of CRC in arterial phase.

Parameter	AUC	95% confidence interval		Standard error			P-value	Optimal threshold		Sensitivity (%))		Specificity (%)
		Upper limit	Lower limit								
NIC	0.920	0.842 0.967		0.029			0.000	0.255		95.7		82.8
K value	0.770	0.668 0.854		0.057			0.000	2.035		65.2		75.0
DEI	0.903	0.821 0.956		0.032			0.000	0.0205		91.3		75.0

**Table 4:** ROC curve analysis of NIC, K Value, and DEI for diagnosing differentiation of CRC in venous phase.

Parameter	AUC	95% confidence interval	Standard error Lower limit	P-value	Optimal threshold	Sensitivity (%)	Specificity (%)
		Upper limit					
NIC	0.874	0.785 0.935	0.038	0.000	0.705	91.3	68.75
K value	0.837	0.742 0.907	0.045	0.000	2.17	82.6	75.0
DEI	0.886	0.799 0.944	0.039	0.000	0.0245	91.3	73.4

## Data Availability

All data generated or analyzed during this study are included in this published article.

## LIST OF ABBREVIATIONS

CRC: Colorectal Cancer
DSCT: Dual-Source Computed Tomography
DECT: Dual-Energy Computed Tomography
NIC: Normalized Iodine Concentration
K: Spectral Curve Slope
DEI: Dual-Energy Index
MDCT: Multi-Detector Computed Tomography
ROI: Region of Interest
ROC: Receiver Operating Characteristic
AUC: Area Under the Curve
CT: Computed Tomography
PACS: Picture Archiving and Communication System

## STANDARDS OF REPORTING

STROBE guidelines were followed.
